# Investigating efficacy of “microbiota modulation of the gut-lung Axis” combined with chemotherapy in patients with advanced NSCLC: study protocol for a multicenter, prospective, double blind, placebo controlled, randomized trial

**DOI:** 10.1186/s12885-021-08448-6

**Published:** 2021-06-22

**Authors:** Qing Xia, Guojie Chen, Yanbei Ren, Tiansheng Zheng, Changxing Shen, Ming Li, Xiangyun Chen, Hong Zhai, Zhuang Li, Jianfang Xu, Aiqin Gu, Meiling Jin, Lihong Fan

**Affiliations:** 1 Department of Pulmonary and Critical Care Medicine, Shanghai 10th People’s Hospital, Tongji University School of Medicine, No. 301, Middle Yangchang Rd, Shanghai, 200072 China; 2 Institute of Energy Metabolism and Health, Tongji University School of Medicine, No. 301, Middle Yangchang Rd, Shanghai, 200072 China; 3 Oncology Department, Shanghai Pulmonary Hospital, Tongji University School of Medicine, No.507, Zhengmin Rd, Shanghai, 200433 China; 4 Oncology Department, Shanghai Chest Hospital, Shanghai Jiaotong University, No.241, West Huaihai Rd, Shanghai, 200030 China; 5 Department of Pulmonary and Critical Care Medicine, Shanghai Zhongshan Hospital, Fudan University School of Medicine, No.180, Fenglin Rd, Shanghai, 200032 China

**Keywords:** Non-small cell lung cancer, Gut-lung axis microecology, Chemotherapy, Probiotics

## Abstract

**Background:**

Most NSCLCs metastasised out of the lungs at the time of diagnosis and cannot be surgically removed . Cytotoxic chemotherapy drugs have become the main treatment in recent decades, especially in patients with NSCLC without EGFR, ALK, and ROS gene mutations. The prognosis of lung cancer is poor, and the overall 5-year survival rate is only 9–13%. Therefore the treatment of advanced NSCLC remains a significant medical need. Recent studies have shown a significant relationship between the gut-lung axis microecology and malignant tumors. Intestinal probiotics are likely to play a role in inhibiting tumorigenesis through “intestinal-pulmonary axis microecological regulation”. This study will seek to investigate the efficacy of “Microbiota modulation of the Gut-Lung Axis” combined with chemotherapy in patients with advanced NSCLC.

**Methods:**

The research is a multicenter, prospective, double blind, placebo controlled, randomized trial. Based on the theoretical basis of “intestinal and lung axis microecological adjustment”, combined with traditional platinum-containing two-drug chemotherapy, the efficacy of the new therapy on patients with advanced NSCLC was observed. Collect the basic information of the patient, and study the effect of platinum-based combined chemotherapy on the diversity of intestinal flora in patients with lung cancer after receiving chemotherapy treatment, feces before and after chemotherapy, and the status and extent of adverse reactions during chemotherapy . A total of 180 subjects were included, divided into a control group (platinum-containing dual-drug chemotherapy) and an intervention group (platinum-containing dual-drug chemotherapy combined with Bifico), and were randomly assigned to the group 1:1.

**Discussion:**

As a result, intestinal-pulmonary microecological balance could become a new target for the treatment of lung cancer. This study explores the combination of intestinal microecological regulation and chemotherapy to provide new treatment strategies and basis for lung cancer patients. It can help prolong the survival time of lung cancer patients and improve the quality of life, thereby generating huge economic and social benefits. The results can be promoted and applied to units engaged in the treatment of lung cancer.

**Trial registration number:**

NCT03642548, date: August 22, 2018, the first version protocol. The URL of trial registry record: https://clinicaltrials.gov/ct2/show/NCT03642548?term=NCT03642548&draw=2&rank=1.

## Background

### Epidemiology of non-small cell lung cancer

Lung cancer is one of the malignant tumors with the highest morbidity and mortality in the world. The increasing incidence of lung cancer in my country is the main cause of the death of malignant tumors [1]. Among the kinds of lung cancer, non-small cell lung cancer (NSCLC) accounts for about 85% of the total number of lung cancers.

### The present status of treatment and technology of advanced non-small cell lung cancer

Most NSCLCs have been locally advanced or distantly metastatic at the time of treatment and cannot be surgically removed. Cytotoxic chemotherapy drugs have been the main treatment in recent decades [2], especially in patients with NSCLC without EGFR, ALK, and ROS1 gene mutations. The current standard chemotherapy for lung cancer is based on platinum-based dual-agent chemotherapy, such as AP, GP, PC, EP, NP, etc., but the objective response rate of chemotherapy is only 25–30%, and bone marrow transplantation, nausea and vomiting, constipation and other adverse reactions are obvious. The prognosis of advanced lung cancer has not been greatly improved, and the overall 5-year survival rate of lung cancer patients is only 9–13%. Therefore, the treatment of advanced NSCLC remains a far-reaching medical need.

### Relationship between gut-lung axis microecology and malignant tumors

In recent years, a very close relationship between the gut-lung axis microecology and malignant tumors have been found in some studies. The large number of intestinal flora is called the second largest gene pool in humans. Its distribution, products and functional metabolism have a profound impact on the host by regulating the host’s nutrient absorption and biochemical indicators, especially the immune system. In 2016, the US White House announced the launch of the “National Microbiome Program” (NMI). The program explored the impact of microbial community abundance in the human body, thereby helping us understand its role in human health and diseases (https://obamawhitehouse.archives.gov/blog/2016/05/13/announcing-national-microbiome-initiative). In April 2017, an important document published by Nature magazine proposed “micro-ecological regulation of intestinal-pulmonary axis”, which confirmed that intestinal flora is closely related to tumors [3–9].

With the advancement of metagenomics principles and high-throughput sequencing technologies, continuous researches have found that the lung microecological balance is inextricably linked to the host immune response. An article published in Nature Medicine by the Dickson team at the University of Michigan in April 2017 revealed that the microorganisms in the lungs are not the only pathogens of pneumonia, but also intestinal microorganisms are able to diseases in the lungs [10, 11]. In the last decade, clinicians and scientists have been paying considerable attention to the study of the “intestinal-pulmonary axis”. Since 2015, major clinical trials aimed at verifying the ability of intestinal bacteria to cause lung pathology. Two of the trials gave intestinal beneficial probiotic bacteria, and the other experiment was given narrow-band antibiotics, designed to specifically change the intestinal microbial distribution and affect the “gut-lung axis” [10].

Although tumors are considered to be caused by both genetics and environmental factors, 20% of human malignant tumors are associated with microorganisms [12]. The microbiome generally causes cancer to develop through the following three pathways: (1) A change in the balance of host cell proliferation and death; (2) Regulation of the innate and adaptive immune function; (3) and by affecting the metabolism of host factors [13]. There is also a close relationship between lung cancer and the microbiome. Studies have confirmed that the oral microbiome is associated with an increased risk of lung cancer [14]. Pulmonary infection with *Mycobacterium tuberculosis* is also noted to increase the risk of patients to lung cancer [15]. Evidence from numerous studies suggests that intestinal microecology can affect lung immunity and microecological stability. Host epithelial cells and other immune cells can directly regulate the inflammatory response from microorganisms and accompanying local cytokines. This form of immune response often occurs in the lungs and other organs [16–19]. Therefore, there is a close relationship between intestinal microecology and that of lung microecology. Changes in intestinal microflora could cause changes in lung immunity and microecology.

### Effects of probiotics on intestinal microecology and immunity

Probiotics are kind of active microorganisms that are beneficial to the host. Probiotics refer to the microorganisms that are beneficial to the host. Probiotics refer to the microorganisms that colonize the human intestine and other organs and function to regulate and keep the microecological balance of the host. They include but are not limited to Bifidobacterium, Lactobacillus, and Clostridium butyricum.. Among them, Bifidobacterium is an important probiotic that has been widely researched [20]. It produces corresponding dynamic changes with age, diet, disease, drugs, etc. in type and quantity. With the increase of age, the number is decreasing. The anti-tumor mechanisms of Bifidobacterium include: (1) Significantly increasing the activity of glutathione-S-transferase and reduce the toxicity of toxic metabolites and carcinogens; (2) Change the metabolism of intestinal spoilage bacteria, such as azo reductase and nitroreductase, and inhibit the catalytic release of carcinogenic precursors in the intestine; (3) Regulate the body’s immune response, activate the differentiation and reproduction of T cells and B cells, increase the number of lymphocytes in the blood, and activate the functions of macrophages, NK cells, MHC II + cells and other immune cells, thereby activating the body Immune Function; (4) Reduce the growth of carcinogenic potential harmful bacteria. In addition, Bifidobacterium can regulate the immunotherapy of tumors. The anti-tumor effect of oral Bifidobacterium in animal experiments is equivalent to PD-1, and the combination of Bifidobacterium and PD-1 can almost completely inhibit the development of tumors [21]. At present, the prevention and treatment of tumors by Bifidobacterium has gradually deepened at the molecular level. Hoarau et al. found that Bifidobacterium can inhibit the phosphatidylinositol 3-kinase (PI3K)/protein kinase B (Akt) signaling pathway and regulate the function of dendritic cells [22]. Huang et al. found that Bifidobacterium can inhibit the inflammatory response in Caco-2 cells infected with Salmonella by inhibiting PI3K/Akt signal, thereby exerting an anti-infective effect [23]; Wang et al. found that Bifidobacterium can down-regulate the expression of survivin in colon cancer LoVo cells by inhibiting PI3K/Akt signal transduction pathway, and up-regulate the expression of p53, and ultimately promote apoptosis.

The mouse lung cancer model confirmed that feeding mice with three mixed antibiotics of vancomycin, ampicillin, and neomycin disrupted the balance of intestinal flora. After being fed with bifidobacteria, the mice which were treated with cisplatin monotherapy compared with the control group, showed a significant reduction in tumor [24], which suggests that Bifidobacterium participates in the systemic anti-lung cancer response, and the anti-tumor effect of probiotics could be manifested in lungs. Current researches on the intestinal-pulmonary axis focus mainly on the changes of lung immune cells. In the study of allergies and inflammations such as asthma, it is believed that short-chain fatty acids (products of intestinal probiotics that decompose dietary fiber), SCFAs) inhibits the differentiation of primitive T cells into Th2 cells [25] and promotes the differentiation of primitive T cells into Treg cells, in order to maintain a healthy immune system. Excessive activation of Th2 cells can trigger the release of cytokines as well as allergic antibodies, and weaken the immune tolerance of Treg cells. Researchers are therefore exploring the possibility of inhibiting the number of Th2 cells in the lungs and increasing the number of Tregs by regulating a balance using intestinal flora. It is known that intestinal probiotics can upregulate the levels of Treg and regulatory dendritic cells, thereby promoting the expression of regulatory cytokines TGF-β and IL-10 and reversing the tumor process [26].

This shows that intestinal probiotics have a role in inhibiting tumorigenesis through “intestinal-pulmonary axis microecological regulation”.

Through basic research and clinical observation, we continue to recognize the importance of the “gut-lung axis” in the regulation of lung cancer. We believe that the imbalance of intestinal flora may provide a pathological basis for the occurrence of lung microecological imbalances, and probiotics are essential for maintaining the balance of intestinal flora. If this hypothesis is proven to be true, the intestinal-pulmonary micro ecological balance will become a new target for the treatment of lung cancer, thereby prolonging the survival period of patients with advanced lung cancer and improving their quality of life. Economically, it will reduce financial burdens on patients in terms of medical bills and saving medical resources as a whole.

## Methods

### Main research objectives

Based on the theoretical basis of “intestinal and lung axis microecological adjustment”, combined with traditional platinum-containing two-drug chemotherapy, the efficacy of the new therapy on patients with advanced NSCLC was observed. Collect the basic information of the patient, and study the effect of platinum-based combined chemotherapy on the diversity of intestinal flora in patients with lung cancer after receiving chemotherapy treatment, feces before and after chemotherapy, and the status and extent of adverse reactions during chemotherapy .

### Research design

## Comparison method and grouping method

A total of 180 subjects were included, divided into a control group (platinum-containing dual-drug chemotherapy) and an intervention group (platinum-containing dual-drug chemotherapy combined with peficon), and were randomly assigned to the group 1:1. (Shanghai Tenth People’s Hospital center account for 40 cases, Shanghai Pulmonary Hospital account for 70 cases, and Shanghai Chest Hospital account for 70 cases).

## Intervention

Both the control group and the intervention group received platinum-based doubletsp chemotherapy. Control group: platinum-based doubletsp + placebo; intervention group: platinum-based doubletsp + Bifico.

(1) Dose selection/adjustment: For the dosing regimen of platinum-based doubletsp chemotherapy used in this trial, please refer to the approved product instructions. Bifico 2 capsules (420 mg) orally three times a day, placebo 2 capsules orally three times a day.

(2) Administration time: platinum-based doubletsp chemotherapy is given every 3 weeks for a total of 4 cycles; administration of Bifico until the death of the patient or the end of follow-up.

(3) Blind/unblind trial: Double blind, generating random numbers to prepare blind bases, randomize drugs, assign patients into groups for medication, investigators record test results and make efficacy evaluations, monitors perform inspections, data management, and statistical analysis. The entire process must be blinded.

### Ethical approval

The study has been approved by the Ethics Committee of Shanghai Tenth People’s Hospital, approval number: SHSY-IEC-KY-4.0/18–177/01.

### Main observation indicators

Objective response rate, ORR.

### Secondary observation index

Progression-free survival PFS, Overall survival time OS, side effects during chemotherapy (gastrointestinal reactions, bone marrow suppression, constipation, etc.), intestinal microecological changes before and after chemotherapy.

### Exploratory index

Fecal metabolomics, blood metabolomics, transcriptomics, proteomics.

### Sample size

This test is designed for superior efficiency, and ORR is the primary endpoint. Study α = 0.05. When the research group predicted ORR = 0.41, the power was 0.85, α = 0.05 (both sides), the control group ORR was 0.25, the expected sample size for each group was 72 cases, considering the drop-out rate of 20%, it was planned to require 90 cases per group. The sample is 180 cases.

### Eligibility criteria

#### Inclusion criteria

(1) Patients with advanced non-small cell lung cancer diagnosed for the first time; (According to the International Lung Cancer Research Association and the American Joint Committee on Cancer Classification 8th Edition TNM staging of lung cancer, with histological or cytological confirmation of locally inoperable (stage IIIB/IIIC), metastatic, inoperable and unable to undergo radical concurrent chemoradiotherapy Or patients with recurrent (stage IV) NSCLC);

(2) No previous anti-tumor therapy for advanced diseases;

(3) Age ≥ 18 years old and ≤ 75 years old;

(4) Accept platinum-containing dual-drug chemotherapy regimen;

(5) PS (ECOG) score: 0 ~ 1 points; expected survival period is more than 3 months;

(6) The function of main organs is normal, that is, it meets the following standards:

1) Good hematopoietic function, defined as white blood cell count ≥3.0 × 10^9^/L, absolute neutrophil count ≥1.5 × 10^9^/L, platelet count ≥100 × 10^9^/L, hemoglobin ≥90 g/L (without transfusion within 14 days).

2) Biochemical examinations must meet the following standards: BIL < 1.25 times the upper limit of normal value (ULN); ALT and AST < 2.5ULN; if there is liver metastasis, ALT and AST < 5ULN; Cr ≤ 1.5ULN or creatinine clearance (CCr) ≥60 ml/min; good coagulation function, INR and PT ≤ 1.5 times ULN; if the subject is receiving anticoagulant therapy, as long as PT is within the intended use range of anticoagulant drugs; Women of childbearing age should agree to use contraceptive measures (such as an IUD, birth control pills or condoms) during the study period and within 6 months after the end of the study; Serum or urine pregnancy test is negative within 7 days before study enrollment, and must be a non-lactating patient; Men should agree to patients who must use contraception during the study period and within 6 months after the end of the study period.

(7) Patients voluntarily joined the study, signed informed consent, and had good compliance.

#### Exclusion criteria

 (1) Small cell lung cancer (including lung cancer mixed with small cell carcinoma and non-small cell carcinoma);

(2) Those with multiple factors that affect oral medications (such as inability to swallow, post-gastrointestinal resection, chronic diarrhea, and intestinal obstruction);

(3) Known symptomatic brain metastasis, spinal cord compression, cancerous meningitis, or brain or pia mater disease detected by CT or MRI;

(4) Those with a history of psychotropic substance abuse who are unable to quit or have mental disorders;

(5) Patients participating in other clinical studies;

(6) Patients with autoimmune diseases or immunodeficiency diseases;

(7) Known allogeneic organ transplantation (except corneal transplantation) or allogeneic hematopoietic stem cell transplantation.

(8) Patients with co-existence of other malignant tumors, except those that have been cured such as cervical carcinoma in situ and non-melanoma skin cancer, or concomitant diseases that according to the investigators’ judgment would seriously endanger the safety of the patient or affect the completion of the study.

### Early withdrawal/termination criteria

 (1) Patient who cannot tolerate four cycles of chemotherapy;

(2) Patients who are difficult to complete the baseline test;

(3) Patients who cannot take Peficon regularly and on time;

(4) Patients with serious adverse events;

(5) Patients who respond to the treatment poorly;

(6) Patients who violate the test plan;

(7) Patients who voluntarily request to withdraw from the test.

### Informed consent, screening and signature

Informed consent: understand and know the purpose and method of the study; voluntarily cooperate with the test process; and sign the informed consent.

### Treatment and evaluation plan

**Fig. 1:**
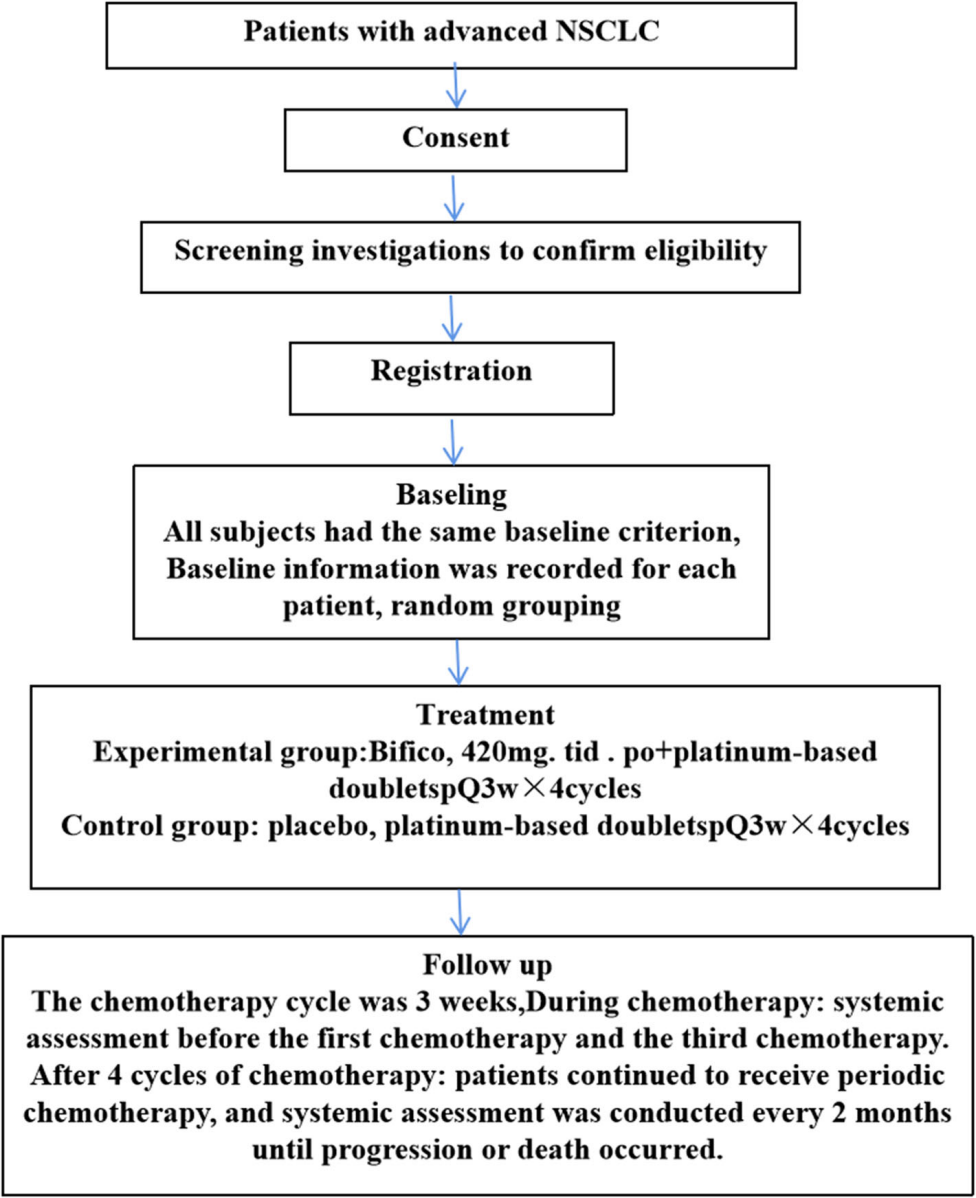
The participant pathway of the clinical trial

After the second and fourth chemotherapy in the course of the study, the efficacy evaluation was carried out, according to RECIST 1.1, the diameter of the target lesion and metastasis were measured, and the change of the disease condition was judged. After 4 times of chemotherapy, the target lesions were followed up once a month, and the complete efficacy evaluation was conducted every 3 months. Followed up until the disease progressed or intolerable toxicity occurred, a total of 5 years of follow-up. Evaluation methods include: physical examination, routine hematology examination, chest enhanced CT, abdominal ultrasound, craniocerebral enhanced MRI and bone scan. Disease control (CR + PR + SD) and adverse reactions can tolerate continued drug use by the patient until the end of the study when the investigator believes that the patient is not suitable for continued medication or when the efficacy evaluation is progressive disease (PD). Simultaneously evaluate safety during medication. Before PD does not appear, other anti-tumor treatments cannot be performed, the participant pathway,see the Fig. 1.

### Statistical analysis

After collecting all clinical data, enter the computer, use Microsoft Access2007 software to establish database data, and use SPSS 22.0 for windows statistical software package for statistical processing after all data is entered. The specific methods of statistical analysis are selected as follows:

(1) Measurement data: the comparison of the mean before and after treatment in each group was by paired t test, and the comparison of the mean between the two groups was by independent sample t test. The *P* value was selected based on the homogeneity test of variance, and the rank sum test between two groups was used for those who did not meet the normal distribution.

(2) Counting data: use χ2 test.

(3) Ordered rank data: use rank sum test.

(5) K-M survival analysis, COX regression.

(5) 0.05 is the statistically significant standard for the two-sided test.

### Test and data management

 1) Overview of data management methods: This experiment uses an electronic data collection system (EDC) for data management. The researcher must ensure that the data is true, complete, and accurate, and no blank or missing items are allowed. When making any corrections, the original records shall not be erased and overwritten, crossed out and marginalized with the revised data, explaining the reason, signature and date. All patients completed the trial, and the original data were all entered into the EDC system. The researchers, statistical analysts, and data managers conducted a blind review of the data. After confirming that the data was not in doubt, the parties signed the database lock application form, and the data administrator will lock the database. After the database is locked, the data administrator exports and analyzes the database and hands it to the statistician for statistical analysis.

2) Report and collection of adverse events and serious adverse events: detailed records of the name of the adverse event, the specific time, severity, whether to take measures, the impact on the research product, the outcome, and whether to withdraw from the test due to the adverse event.

3) Medical safety measures: strictly regulate medication and try to avoid adverse events.

4) Communication with the ethics committee and the higher-level drug supervision department: timely and regular communication.

5) Internal analysis plan of the data: the researcher will analyze and review the test data.

6) Frequency of submitting data safety and monitoring reports to the ethics committee: at least 1–2 times per month.

## Discussion

Lung cancer is the malignant tumor with the highest morbidity and mortality in my country. It is therefore practically significant to make changes and to enrich the treatment mode of lung cancer. The study found that there is a very close relationship between intestinal microecology and malignant tumors. The distribution, products and functional metabolism of intestinal flora have an important impact on the immune system of patients with NSCLC. Base on evidence from the intestinal-pulmonary axis in lung cancer regulation, intestinal microflora imbalance are thought to be partly responsible (or responsible) in the occurrence of lung microecological imbalances. As a result, intestinal-pulmonary microecological balance could become a new target for the treatment of lung cancer. This study explores the combination of intestinal microecological regulation and chemotherapy to provide new treatment strategies and basis for lung cancer patients. It can help prolong the survival time of lung cancer patients and improve the quality of life, thereby generating huge economic and social benefits. The results can be promoted and applied to units engaged in the treatment of lung cancer.

### Clinical trial status

At the time of submission, the study is ongoing and open to recruitment patients.

**Trial Registration Number:NCT03642548, date: August 22, 2018.**

## Data Availability

Not applicable.

## Abbreviations

NSCLC: non-small cell lung cancer
EGFR: epidermal growth factor receptor
ALK: Anaplastic lymphoma kinase
AP: alimta + platin
GP: gemcitabine + platinum
PC: platinum + cyclophosphamide
EP: etoposide + platinum
NP: navelbine + platinum
SCFAs: short chain fatty acids
PFS: progression-free survival,
OS: overall survival time
ORR: Objective Response Rate
PS: performance status
PD: progressive disease
PR: partial response
CR: complete response
SD: stable disease
